# Acute on Chronic Heart Failure Secondary to Left Ventricular Noncompaction

**DOI:** 10.1155/2020/6369806

**Published:** 2020-10-26

**Authors:** Ivana Capin, Christine A. Capone, Matthew D. Taylor

**Affiliations:** ^1^Mount Sinai Kravis Children's Hospital, New York, NY, USA; ^2^Cohen's Children's Medical Center, Northwell Health, New Hyde Park, NY, USA

## Abstract

Left ventricular noncompaction (LVNC) is a rare cardiomyopathy characterized by hypertrabeculations and intertrabecular recesses most often seen in the left ventricle (LV). The patient may be asymptomatic or present with heart failure, arrhythmia, and sudden death. We discuss a previously healthy 7-year-old male who presented to the Emergency Department (ED) multiple times over a three-week period. His complaints evolved over the course of his illness, initially presenting with fatigue and suicidal ideation, followed by diffuse abdominal pain. Prior to his ICU admission, he had been discharged from the ED twice, due to well appearance and reassuring lab findings. He returned to the ED a final time with severe venous congestion and cardiogenic shock with acute hepatic injury. Echocardiogram revealed LV apical hypertrabeculation with a severe dilated cardiomyopathy and biventricular failure along with a large thrombus in the left ventricular cavity. Congestive heart failure and anticoagulation therapy was initiated, and the patient went on to biventricular assist device (BiVAD) placement and cardiac transplant. Although LVNC is rare, pediatric heart failure does present to the general pediatrician and has high morbidity and mortality. The presenting symptoms can be obscure and pose a challenge to pediatricians. This case report and review will assist in familiarizing the general pediatrician with pediatric heart failure presentation, treatment, and course.

## 1. Background

Left ventricular noncompaction (LVNC) is a rare cardiomyopathy of both familial and sporadic origin [1, 2]. Familial inheritance is more common in the pediatric population and demonstrates various penetrance and presentation [3]. LVNC is caused by arrest of embryonic endomyocardial morphogenesis leading to the characteristic finding of prominent trabeculations and intertrabecular recesses within the LV cavity [2]. Presentation ranges from incidental diagnosis to patients presenting with cardiogenic shock, thromboembolism, arrhythmia, and sudden death [1, 4, 5]. We report a case of LVNC in a previously healthy 7-year-old male who presented with chronic abdominal pain, vomiting, anorexia, and fatigue complicated by dyspnea, acute liver injury, and shock concerning acute decompensation of chronic heart failure.

## 2. Case Presentation

**Figure 1:**
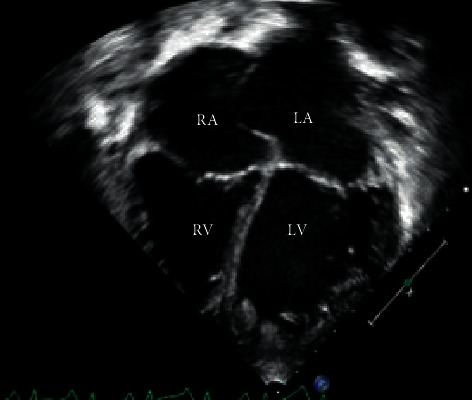
Apical four chamber view showing bilateral atrial and ventricular dilation with anterior bowing of the interatrial septum suggestive of elevated left atrial pressure. There are also prominent hypertrabeculations at the apex of the left ventricle. LA: left atrium. LV: left ventricle. RA: right atrium. RV: right ventricle.

**Figure 2:**
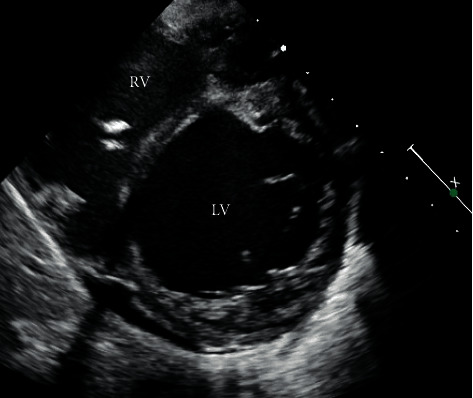
Parasternal short-axis view of the left ventricle revealing hypertrabeculations and deep intertrabecular recesses.

**Figure 3:**
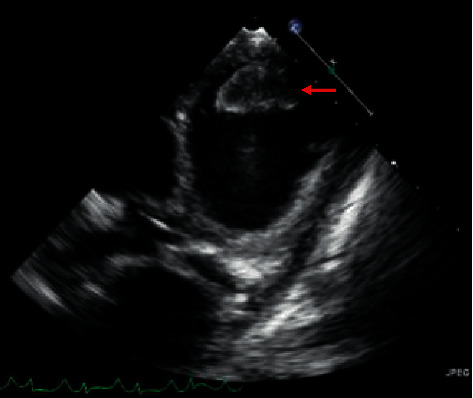
Modified parasternal short-axis view revealing a 2.7 × 1.3 cm thrombus in the left ventricle.

A 7-year-old male with no past medical history presented to his primary practitioner and was referred to emergency care for suicidal ideation, abdominal pain, nausea, vomiting, anorexia, and fatigue. The patient was unable to keep up with his peers, which led to negative thoughts. Vitals at the time were a heart rate (HR) of 124, respiratory rate (RR) of 20, oxygen saturation (SpO2) of 99%, and blood pressure (BP) of 90/70 mm Hg, and the patient was afebrile. The patient had a psychiatric evaluation in a pediatric emergency department (ED) and was provided with reassurance. Two weeks later, the patient again presented to the ED for continued symptoms. His vitals were documented as follows: HR, 127; RR, 24; SpO2, 100%; and BP, 92/58, and he was afebrile. On arrival, he was well appearing, with a soft, nontender abdomen; however, further evaluation was pursued due to the persistent nature of his symptoms and tachycardia of unclear etiology. His complete blood count and comprehensive metabolic panels were normal though alanine aminotransferase and aspartate aminotransferase were slightly above normal limits. An abdominal ultrasound was negative for appendicitis, but equivocal for choledocholithiasis prompting referral to gastroenterology. On follow-up with gastroenterology one week later, he was again referred to the ED due to tachycardia and tachypnea as well as severe abdominal pain and shortness of breath. Physical exam on admission to ICU revealed jugular venous distension, peripheral edema, hepatomegaly, crackles on pulmonary auscultation, and diminished S1, S2, and an S4 gallop. Abdominal X-ray was initially done demonstrating incidental cardiomegaly which led to a chest X-ray confirming an enlarged cardiac silhouette and showing significant pulmonary edema. Based on this, an echocardiogram was performed and revealed severe global hypokinesia of the left ventricle (ejection fraction 14% and shortening fraction 6%) with left ventricular apical hypertrabeculation and severe dilatation (LV end diastolic dimension Z score, 7.99) as shown in Figures 1 and 2. The Jenni criteria were used to make the diagnosis of LVNC. Tricuspid valve regurgitation peak velocity was 2.75 m/s. Mitral valve E/A ratio was 2.14, indicating preserved diastolic function with no evidence of mitral stenosis. Additionally, there was a well-circumscribed echogenic mass (2.7 × 1.3 cm) concerning for a left ventricular thrombus within the apex of the left ventricular cavity as shown in Figure 3. Labs were significant for a chronic, compensated metabolic acidosis 7.42/26/20 with a base deficit of 6.7, a venous lactate of 5.6, and transaminitis with AST and ALT of 1225 and 846, respectively. The patient also had hyperbilirubinemia and was coagulopathic (PTT 38.9 and INR 3.91).

Given the diagnosis of acute on chronic heart failure secondary to LVNC, the patient was started on furosemide, milrinone, and low molecular weight heparin despite the initial coagulopathy due to intracardiac thrombus. Biventricular systolic function continued to worsen, and the patient underwent biventricular assist device placement with removal of clot 6 days following initial presentation to the ICU. He underwent heart transplantation 17 days following initial presentation. He has been discharged home on maintenance medications with an uncomplicated posttransplant course. On follow-up with his mother, the patient has returned to his playful baseline and psychological issues have been resolved.

## 3. Discussion

Left ventricular noncompaction (LVNC) is a rare disorder accounting for approximately 9% of newly diagnosed pediatric cardiomyopathies [6]. It is characterized by prominent trabeculations and intertrabecular recesses within the LV cavity [7–10] caused by the arrest of endomyocardial morphogenesis [7, 9, 10]. LVNC is a disease that affects all age groups, though it is present from birth with varying degrees of severity, leading to variable age at diagnosis [7, 11]. Due to the increasing accessibility of echocardiography, the identification, prevalence, and understanding of LVNC have continued to grow over time. Clinical presentation ranges from asymptomatic individuals diagnosed incidentally to patients presenting with heart failure, cardiogenic shock, thromboembolism, arrhythmia, and sudden death [2].

Although LVNC is rare, our patient's presentation is a common manifestation of pediatric heart failure from any cause. While adults typically present with shortness of breath, pediatric heart failure is much less common and has more heterogeneous symptomatology leading to significant difficulty in diagnosis for primary care practitioners [12, 13].  Table 1 details the signs and symptoms of pediatric heart failure stratified by age group as a reference for the general practitioner. Heart failure symptoms vary from infancy to adolescence. Infants of 0-1 year may have difficulty feeding, failure to thrive, or irritability. Young children of 1–10 years often present with cough, wheeze, or gastrointestinal complaints along with the inability to tolerate activity and are often misdiagnosed with asthma or gastroenteritis [14–16]. Adolescents and young adults primarily present with abdominal pain in isolation or in conjunction with respiratory complaints such as chronic cough. In contrast to adults, children are less likely to present with chest pain, peripheral edema, or palpitations [17]. Notably, gastrointestinal (GI) complaints (failure to thrive, anorexia, abdominal pain, nausea, and vomiting) are a common component of the initial heart failure presentation in children. Isolated GI complaints occurred in 16% of infants, 14% of children, and 23% of adolescents who presented to the emergency room in heart failure [14]. Macicek et al. found that up to 9/10 patients in heart failure had a coexisting GI complaint [15]. The high incidence of GI symptoms may be one reason why heart failure in children is often under-recognized. Although cardiomyopathy is rare, with an incidence of 1–1.5 per 100,000, it carries significant morbidity and mortality; early identification is crucial [18]. Heart failure is often a presenting sign for patients with cardiomyopathy or other cardiac pathologies such as myocarditis. Pediatricians must be cognizant of the signs and symptoms of chronic heart failure and progressive cardiac disease.

Our patient presented with progressive gastrointestinal symptoms. Although he exhibited signs of heart failure prior to this (manifesting as depression from inability to keep up with this peers), his progressive GI complaints were the hallmark of his disease. He, like many others in the literature, was diagnosed with heart failure after abdominal imaging showed a lower margin of an enlarged heart.

The present case reinforces the notion that pediatricians should be highly suspicious of persistent GI complaints in children with or without concomitant respiratory symptoms [14]. In these cases, a detailed history and physical examination are crucial in obtaining the diagnosis; specifically, every examination must ensure an evaluation for tachycardia, pulmonary congestion, hepatomegaly, S4 gallop, and jugular venous distention [16].

Certain lab tests have been helpful adjuncts in the diagnosis and management of pediatric heart failure. Markers such as brain natriuretic peptide (BNP) and pro-BNP have been used to differentiate cardiac disease from respiratory pathology [19]. Hyponatremia (defined as <135 mmol/L) has also been found to assist in the guidance of management of patients with acute decompensated heart failure due to its association with an increased risk of inhospital mortality and requirement of mechanical circulation and transplant [20]. Intracardiac thrombus is a complication of poor cardiac function and must be addressed promptly at a heart failure center due to the risk of pulmonary embolism or stroke.

A heightened awareness of the common patterns for the presentation of pediatric heart failure will aid physicians in earlier recognition, leading to a more timely engagement of cardiologists and the initiation of appropriate therapy. Although heterogeneous in its presentation, LVNC has a relatively high incidence of mortality and transplantation (∼18%) [21]. As such, these patients require close cardiology follow-up in centers specializing in heart failure and cardiomyopathy.

## 4. Conclusion

LVNC is an increasingly recognized cardiomyopathy classically presenting with symptoms of chronic heart failure. Due to the rarity and heterogeneity of presentation of heart failure in children, it can be easily missed in the general pediatric practice or in a busy acute care setting. Our case highlights a child who presented with inability to keep up with his peers and persistent abdominal pain mistaken as gastroenteritis. At the time of diagnosis of the causative LVNC, the patient had progressed to acute, severe decompensated heart failure associated with left ventricular thrombus and acute liver injury. A heightened awareness of the common patterns for presentation of pediatric heart failure will facilitate early diagnosis, referral, and treatment.

## Figures and Tables

**Table 1:** Signs and symptoms of heart failure in the pediatric patient population [14–16].

Age	Symptoms	Signs
Infant (0-1 years)	Difficulty feeding, failure to thrive, irritability, poor weight gain	Tachycardia, tachypnea, diaphoresis, cyanosis, murmurs, respiratory distress
Toddlers and young children (1–10 years)	Failure to thrive, irritability, fatigue, abdominal pain, nausea and vomiting, cough, loss of appetite	Tachycardia, tachypnea, dyspnea, poor air entry, abdominal tenderness, cardiac murmur, jugular venous distention
Adolescents (>10 years)	Shortness of breath, palpitations, anorexia, dizziness, abdominal pain, exercise intolerance, syncope, chest pain	Tachycardia, tachypnea, dyspnea, cardiac murmurs, peripheral edema and cyanosis, jugular venous distention

## Data Availability

No data were used to support this study.

## Abbreviations

LVNC: Left ventricular noncompaction
ICU: Intensive care unit
FTT: Failure to thrive
BNP: Brain natriuretic peptide
BiVAD: Biventricular assist device.
